# Optimization of Bolus-Tracking Thresholds levels in Cerebral CT Angiography: Influence of Patient Characteristics on contrast enhancement dynamics and radiation dose metrics

**DOI:** 10.12688/f1000research.171062.2

**Published:** 2025-11-29

**Authors:** Ashwin Prabhu, Abhimanyu Pradhan, Sharath S, Rajagopal Kadavigere, Winniecia Dkhar, Priya P S, Suresh Sukumar, Pulagam Vamsidhar Reddy, Neil Abraham Barnes

**Affiliations:** 1 Department of Medical Imaging technology, Manipal Academy of Higher Education, Manipal College of Health Professions, Manipal, Karnataka, 576104, India; 2 Department of Radiodiagnosis and Imaging, Kasturba Medical College Manipal, Manipal Academy of Higher Education, Manipal, Karnataka, 576104, India; 3 Fellow in Cardiothoracic Radiology, Narayana Institute of Cardiac Sciences, Bangalore, karnataka, India

**Keywords:** Bolus tracking, CT cerebral angiography, CTDIvol, peak enhancement time, radiation dose

## Abstract

**Background:**

Cerebral computed tomography angiography (CTA) is widely used to assess neurovascular disorders, but venous contamination often obscures arteries. Optimizing bolus-tracking thresholds is crucial, yet patient factors influencing contrast dynamics and the value of radiation dose indices in head CTA remain unclear.

**Objectives:**

To optimize bolus-tracking thresholds in cerebral CTA by examining patient-related influences on enhancement and radiation metrics.

**Methods:**

126 adults undergoing cerebral CTA were evaluated in this prospective study. Demographics, physiologic parameters, peak enhancement time (PET), peak enhancement attenuation (PEA), and dose indices (CTDIvol, SSDE) were recorded. Linear regression identified predictors of enhancement. Two blinded radiologists graded venous contamination. ROC analysis, including age subgroups, determined the optimal HU threshold.

**Results:**

Median age was 55.5 years; 70% were male. PET rose with age (+0.086 s/year, p < 0.001) and was shorter in females (–2.39 s, p = 0.003). PEA increased with threshold (+1.03 HU/unit, p < 0.001). Arterial enhancement was higher in females (+40.7 HU, p < 0.001) and patients ≥60 years (+70 HU, p < 0.001). Venous enhancement correlated with PET (p = 0.023) and systolic pressure (p = 0.002). ROC analysis showed an optimal threshold of 105 ± 5 HU (AUC = 0.634; sensitivity 88.4%, specificity 77.1%). CTDIvol, but not SSDE, correlated with weight (p = 0.015).

**Conclusion:**

Intrinsic (age, gender) and extrinsic (threshold) factors shape CTA enhancement. A 105 ± 5 HU threshold reduces venous contamination, especially in younger patients. CTDIvol remains the preferred dose index. Findings support individualized, resource-efficient CTA protocols aligned with UN SDGs 3, 9, and 12.

## Introduction

Cerebral computed tomography angiography (CTA) has become a fundamental diagnostic tool in neuroimaging, particularly for the rapid evaluation of acute ischemic stroke, intracranial aneurysms, arteriovenous malformations (AVMs), and vascular pathologies requiring surgical or endovascular planning. ^1– 4^ Its widespread availability, fast acquisition, and high-resolution vascular detail make it indispensable in emergency and elective clinical settings. In acute stroke, for instance, CTA is critical for assessing large vessel occlusions and collateral circulation, while in aneurysms and AVMs, it provides essential anatomical mapping for treatment planning. ^5, 6^ Thus, optimisation of cerebral CTA protocols directly impacts diagnostic accuracy and patient outcomes.

Venous contamination represents a critical technical challenge in cerebral CTA, arising from premature venous contrast enhancement that temporally overlaps with the arterial phase. ^7^ This overlap can obscure arterial structures, reduce diagnostic confidence, and sometimes necessitate repeat scans, exposing patients to additional radiation dose and contrast medium. Venous contamination is particularly problematic in posterior circulation imaging, where small arterial branches are easily masked by venous enhancement. Therefore, controlling the timing of image acquisition to maximise arterial opacification while minimising venous overlap is essential to reliable interpretation. ^8^

Current bolus-tracking techniques aim to optimize scan timing by initiating acquisition once intravascular contrast reaches a predefined attenuation threshold, typically measured in Hounsfield Units (HU). ^9^ However, the effect of patient-related factors such as age, gender, body habitus, and cardiac-related factors (heart rate and blood pressure) on peak enhancement time (PET) and peak enhancement attenuation (PEA) remains underexplored. Previous studies have suggested that males may exhibit longer PET due to higher blood volume, and older patients may demonstrate delayed enhancement due to reduced cardiac output. ^10, 11^ Yet, these influences have not been consistently integrated into protocol design. Similarly, the optimal HU threshold for bolus tracking in cerebral CTA has often been based on empirical practice or vendor defaults rather than systematic validation.

Beyond contrast timing, protocol optimisation must also account for radiation dose exposure. ^12– 14^ With growing emphasis on personalized imaging, the size-specific dose estimate (SSDE) has been promoted as a more patient-tailored dose metric than the traditional volumetric CT dose index (CTDIvol). ^15^ While SSDE correlates well with body habitus in chest and abdominal CT, its utility in cerebral CTA is uncertain. The relatively rigid cranial anatomy and consistent skull attenuation may limit the value of SSDE in the head, raising the possibility that CTDIvol remains a more stable and clinically relevant metric for neuroimaging dose assessment.

These gaps highlight the need for a comprehensive analysis integrating contrast dynamics (PET and PEA) and radiation dose behaviour (CTDIvol vs SSDE) in cerebral CTA. Such an approach may allow for better protocol optimisation and evidence-based standardisation of bolus-tracking thresholds to reduce venous contamination across diverse patient populations.

The objective of this study was to optimize bolus-tracking thresholds in cerebral CT angiography by evaluating the influence of patient factors on contrast enhancement dynamics and radiation dose metrics.

## Materials and methods

### Study design and setting

This prospective, single-center observational study was conducted in the Department of Radiodiagnosis and Imaging, Kasturba Hospital, Manipal, India.

### Ethics and registration

Ethical approval for the study was secured from the Institutional Ethics Committee (IEC approval number: IEC2:166/2023). The study was registered with the Clinical Trials Registry–India (CTRI; registration number: CTRI/2023/07/054814), with the registration date being July 5, 2023. Written informed consent was obtained from all participants.

### Participants

**Inclusion criteria:** Adults (≥18 years) referred for clinically indicated CT cerebral angiography (CTA).

**Exclusion criteria:** Patients with recent head trauma or road traffic accidents, inability to cooperate, elevated serum creatinine (>1.5 mg/dL), or known contraindication to iodinated contrast.

**Sampling:** Consecutive eligible patients were enrolled using convenience sampling during the study period. The minimum sample size was calculated (n = 110) using the formula to test the linear regression correlation coefficient. To account for exclusions and variability, 126 participants were ultimately included.

### CT angiography acquisition protocol

Demographic and physiologic variables (age, gender, height, weight, heart rate, and systolic/diastolic blood pressure) were noted before scanning. Body mass index (BMI) was calculated as weight (kg)/height (m ^2^), and renal function was confirmed within institutional policy time windows.

A low-osmolar non-ionic iodinated contrast agent Omnipaque ^®^ 300 (Iohexol 300 mg I/mL, catalogue number-00407141363, manufactured by GE Healthcare) was administered using a dual-head power injector via an Intravenous (IV) cannula. The injection protocol was standardized at 80 mL with a flow rate of 4 mL/s, followed by a saline (40 mL) flush. Patients with elevated creatinine were excluded from analysis; this study did not apply the alternative protocol using Iodixanol (320 mg I/mL).

All scans were performed on a 128-slice multidetector CT system with the patient supine and head-first. Following a non-contrast scan and localizer, bolus tracking was performed at the C4–C5 level by placing a circular ROI in the common carotid artery. Arterial-phase acquisition was auto-triggered when attenuation reached 100–150 HU, with a fixed post threshold delay of 3.6 seconds. Helical coverage extended from the aortic arch to the cranial vertex. A standardized 12-second delayed phase was also acquired for venous comparison. Post-processing was performed on a dedicated workstation. Detailed acquisition parameters are listed in Table 1.

**Table 1.** Scan protocol.

Protocol name	CT cerebral angiography
Position	Supine (head first)
Area coverage	Vertex to Arch of Aorta
Scan direction	Caudocranial
Start location	Arch of Aorta
End location	Vertex
kVp	120
mAs	220
Acquiring images	5x5 mm
Recon	Slice thickness: 1.5 mm Increment: 0.75 mm
Locator/Tracker	Carotid artery (at the level of C3)
Contrast volume	80mL contrast, 40 mL saline at a flow rate of 4 mL/sec
Post threshold delay	Angio phase- 3.6 secs Delayed phase-12 secs

### Quantitative analysis

To measure attenuation (HU), standardized elliptical ROIs (~2–5 mm where feasible, avoiding calcifications and artifacts) were placed in the following vascular structures: basilar artery, right and left middle cerebral arteries (MCA), anterior cerebral arteries (ACA), posterior cerebral arteries (PCA), superior sagittal sinus, inferior sagittal sinus, and bilateral external jugular veins. PET: Time from injection start to the bolus-trigger event on the time–attenuation curve. PEA: Maximum HU reached in the carotid ROI before scan initiation. Scanner-reported dose indices CTDIvol and SSDE were recorded directly from the console.

### Qualitative venous enhancement scoring

Two experienced radiologists (blinded to each other’s results) independently assessed venous enhancement in the angiographic phase. A 3-point Likert scale was used:

1 = minimal venous enhancement,
2 = partial venous enhancement,
3 = maximum venous enhancement.

### Statistical analysis

Analyses were performed using Jamovi software. Normality was assessed with the Shapiro–Wilk test. Continuous variables were expressed as mean ± SD (normally distributed) or median (IQR) (non-normal). Group comparisons used independent-samples t tests (parametric) or Mann–Whitney U tests (nonparametric). Associations between PET/PEA and candidate predictors (age, gender, BMI/weight, systolic/diastolic blood pressure, and heart rate) were tested using multivariable linear regression with multicollinearity diagnostics (variance inflation factor <5). Regression results are reported as β coefficients, 95% confidence intervals (CI), and p values. Arterial and venous attenuation values (HU) were modelled separately against the same predictor set plus PET/PEA. Inter-observer agreement for venous enhancement scoring was assessed with Cohen’s Kappa and 95% CI. A receiver operating characteristic (ROC) analysis with the Youden index was used to identify the optimal attenuation trigger that discriminates between low venous contamination (score = 1) and higher contamination (scores = 2–3). As an additional exploratory analysis, age-stratified ROC curves were generated to evaluate the diagnostic performance of attenuation thresholds across different patient groups. Statistical significance was defined as two-sided p < 0.05.

## Results

### Participant characteristics

A total of 126 participants were enrolled, with a median age of 55.5 years (IQR: 43.3–66). The cohort included 88 males and 38 females. Median weight was 63.5 kg (IQR: 55.3–70), and median BMI was 23.4 kg/m ^2^ (IQR: 21–26.2). Height was normally distributed (mean 164 ± 9.75 cm). Median heart rate was 78 bpm (IQR: 72–86), systolic blood pressure was 140 mmHg (IQR: 130–150), and diastolic blood pressure was 85 mmHg (IQR: 80–90). Median PET was 18 s (IQR: 16–21), and median PEA was 146 HU (IQR: 129–167). The median CTDIvol was 30.2 mGy (IQR: 27.7–30.9), and the median SSDE was 27.6 mGy (IQR: 24.7–30.2) ( Table 2).

**Table 2.** Descriptives of participants’ characteristics.

Parameters	Mean
Age	55.5 (43.3, 66)
Height	(164 ± 9.75) *
Weight	63.5 (55.3, 70)
BMI	23.4 (21, 26.2)
Heart rate	78 (72, 86)
Systole	140 (130, 150)
Diastole	85 (80, 90)
PET	18 (16, 21)
PEA	146 (129, 167)
Threshold	120 (110, 133)
CTDI	30.2 (27.7, 30.9)
SSDE	27.6 (24.7, 30.2)

* Represents the normally distributed data reported in Mean ± SD, whereas others are not normally distributed data reported as median (quartile).

### Factors affecting PET

Correlation and regression analyses showed that age and gender were the most significant determinants of PET. PET increased significantly with age (ρ = 0.312, p < 0.001). Multivariable regression confirmed that each additional year of age delayed PET by +0.086 s (Estimate = 0.0859, SE = 0.0243, t = 3.53, p < 0.001). Gender was also an independent predictor, with females exhibiting significantly shorter PET than males by approximately 2.39 seconds (Estimate = –2.3919, SE = 0.7949, t = –3.01, p = 0.003) ( Table 3).

**Table 3.** Linear regression model coefficients showing the factors affecting peak enhancement time & peak enhancement attenuation.

Predictor	Estimate	Standard error	t- Statistics	p-value
**PET (Dependent Variable)**				
**Intercept**	14.9322	1.3751	10.86	<.001
**Age**	0.0859	0.0243	3.53	<.001
**Gender**	-2.3919	0.7949	-3.01	0.003
**PEA (Dependent Variable)**				
**Intercept**	-12.2416	21.6023	c	0.572
**Threshold**	1.0324	0.1445	7.144	<.001

### Factors affecting PEA

PEA: Was primarily determined by technical bolus-tracking factors rather than patient demographics. Each unit increase in the trigger threshold raised PEA by +1.03 HU (p < 0.001). PET itself did not independently affect PEA ( Table 3).

### PET variations based on age groups

**Figure 1.**
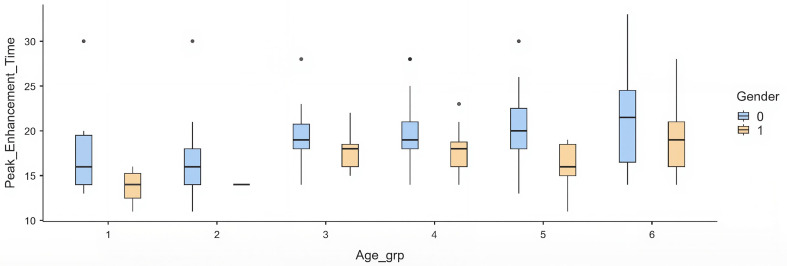
Box plots showing PET distribution across age groups stratified by gender (blue: male, yellow: female). Each box represents the interquartile range, with the median marked by a horizontal line and whiskers indicating variability outside the upper and lower quartiles. Outliers are plotted as individual points. Females consistently demonstrate shorter PET values across all age groups compared to males.

When stratified into age categories, a progressive delay in PET was observed across older groups. Compared with the youngest group (≤30 years), patients aged ≥70 years had a mean delay of +4.1 s (p = 0.012). Intermediate groups (40–60 years) showed modest but non-significant prolongation, while the >60 group had the most substantial effect ( Table 4) ( Figure 1).

**Table 4.** Linear regression model coefficients showing age related variations in peak enhancement attenuation.

Predictor	Estimate	SE	t	p
Intercept ^a^	16.455	1.29	12.803	<.001
Age_grp:				
2 – 1	0.279	1.69	0.165	0.869
3 – 1	2.403	1.59	1.514	0.133
4 – 1	2.900	1.50	1.939	0.055
5 – 1	2.856	1.51	1.892	0.061
6 – 1	4.124	1.61	2.554	0.012

^a^ Age groups were categorized as Group 1 = 20–30 years, Group 2 = 31–40 years, Group 3 = 41–50 years, Group 4 = 51–60 years, Group 5 = 61–70 years, Group 6 = 71–80 years. Group 1 (20–30 years) was used as the reference category in the regression model.

### Factors affecting mean arterial and venous enhancement

**Figure 2.**
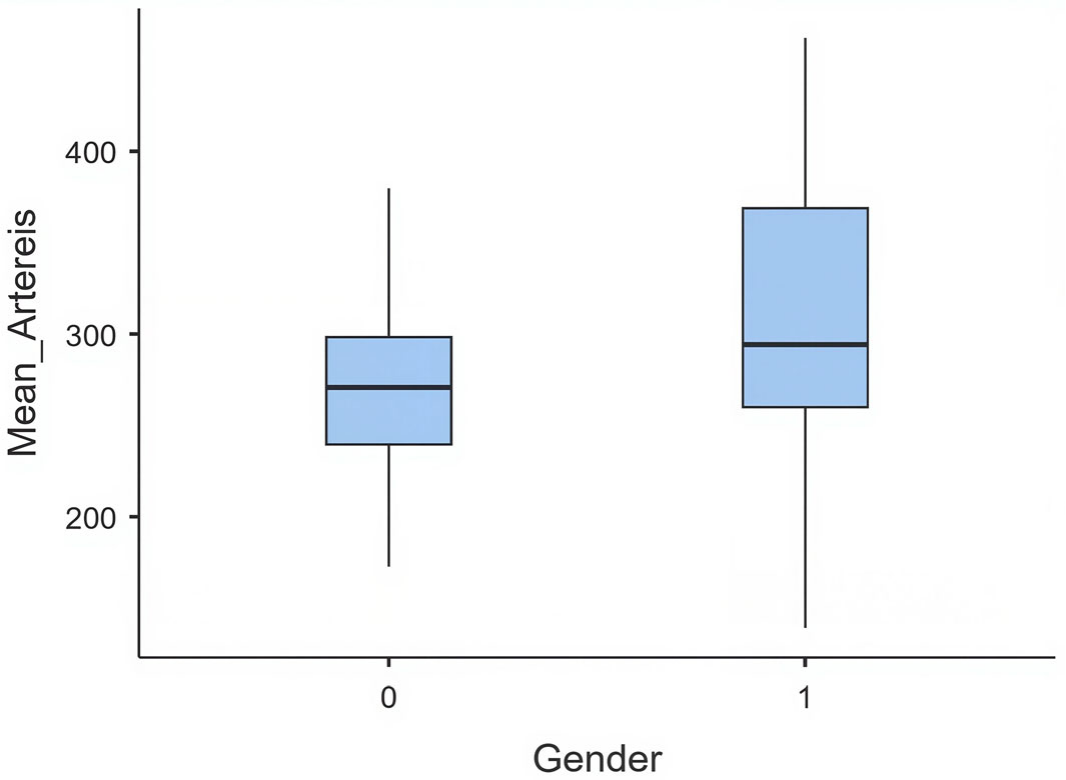
Box plot comparing Mean Arterial Enhancement (Mean_Arteries) between genders (0 = male, 1 female).

Arterial enhancement:

Regression models revealed that arterial enhancement was higher in females than in males (+40.7 HU, p < 0.001), likely reflecting larger blood volume and higher cardiac output. Patients aged ≥60 years demonstrated significantly greater arterial enhancement than the youngest age group (+70 HU, p < 0.001). PET showed a positive but non-significant trend toward higher arterial HU (+1.9 HU per second, p = 0.063) ( Table 5) ( Figure 2).

**Table 5.** Linear regression model coefficients showing factors affecting mean arterial enhancement.

Predictor	Estimate	SE	t	p
Intercept ^a^	239.4738	56.811	4.215	<.001
Gender	40.6949	9.720	4.187	<.001
Threshold	-0.0438	0.339	-0.129	0.898
Peak_Enhancement_Time	1.9271	1.030	1.870	0.064
Age_grp:				
2 – 1	16.3574	18.491	0.885	0.378
3 – 1	13.4326	17.351	0.774	0.440
4 – 1	18.4978	16.449	1.125	0.263
5 – 1	38.9890	16.649	2.342	0.021
6 – 1	70.7001	18.362	3.850	<.001

Venous enhancement:

Regression models revealed that PET strongly influenced venous enhancement (β = 1.77, p = 0.023), meaning that delayed arterial peaks allowed more venous filling. Systolic BP was positively associated with venous HU (β = 0.50, p = 0.002), suggesting faster systemic circulation in hypertensive states. Diastolic BP had an inverse effect (β = –0.88, p = 0.004), potentially reflecting altered venous return dynamics. Neither gender nor heart rate significantly affected venous HU ( Table 6).

**Table 6.** Linear regression model coefficients showing factors affecting mean venous enhancement.

Predictor	Estimate	SE	t	p
Intercept	43.3823	42.325	1.025	0.308
Peak_Enhancement_Time	1.7653	0.768	2.300	0.023
Systole	0.5049	0.163	3.095	0.002
Diastole	-0.8820	0.301	-2.928	0.004
Age_grp:				
2 – 1	10.2849	13.776	0.747	0.457
3 – 1	-15.3341	12.927	-1.186	0.238
4 – 1	-7.0282	12.254	-0.574	0.567
5 – 1	-31.4176	12.404	-2.533	0.013
6 – 1	-22.5185	13.680	-1.646	0.103

### ROC analysis

ROC analysis yielded an AUC of 0.634, indicating fair discriminatory performance. The optimal threshold was 105 ± 5 HU, achieving a sensitivity of 88.4% and a specificity of 77.1%. Thresholds above 120 HU were associated with frequent venous contamination due to delayed triggering and overlap of venous with arterial enhancement. In contrast, thresholds below 100 HU risked premature triggering and inadequate arterial opacification. Thus, a threshold of 105 ± 5 HU appears best for minimizing venous contamination without compromising arterial contrast.

### Age-stratified ROC analysis (Exploratory analysis)

**Figure 3.**
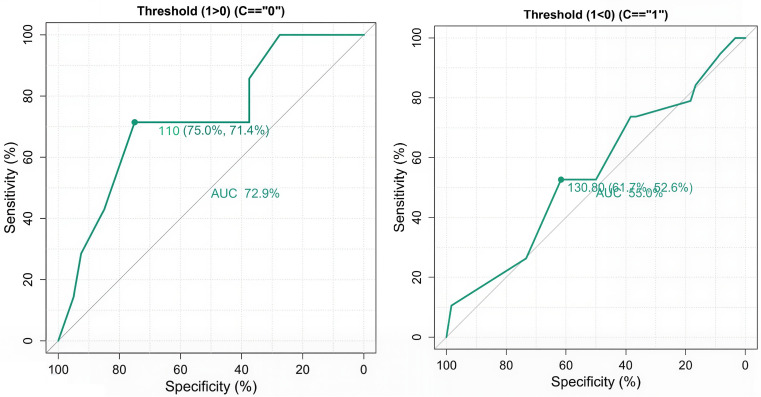
ROC curves comparing diagnostic performance across age groups. A represents the AUC in younger adults, and B represents the AUC in older adults.

An additional exploratory analysis of age-stratified ROC analysis further demonstrated differential diagnostic performance. Among younger adults (<50 years), a threshold of 110 ± 5 HU achieved moderate accuracy (AUC = 0.729), with sensitivity and specificity of 75.0% and 71.4%, respectively. In contrast, for older adults (≥50 years), a threshold of 130 ± 5 HU provided poor discrimination (AUC = 0.55), with lower sensitivity (61.7%) and specificity (52.6%). These findings suggest that HU thresholds are more reliable in younger patients. At the same time, their diagnostic utility diminishes with age, underscoring the need for age-adjusted trigger strategies to enhance clinical accuracy ( Figure 3).

### Inter-observer agreement

Venous contamination scoring demonstrated moderate agreement between the two independent radiologists, with Cohen’s κ = 0.581 (95% CI 0.46–0.70, p < 0.001). This indicates consistent grading across observers, though some variability remained in borderline cases.

### Radiation dose analysis (CTDIvol vs SSDE)

CTDI (CTA) demonstrated a significant positive association with patient weight (Estimate = 1.26, SE = 0.51, t = 2.46, p = 0.015), indicating that higher body weight was consistently associated with higher CTDI values. In contrast, SSDE was not significantly related to patient weight (Estimate = –5.67×10 ^−4^, SE = 3.94×10 ^−4^, t = –1.44, p = 0.153), and its values showed greater variability across patients ( Table 7).

**Table 7.** Linear regression model coefficients showing the better dose estimator in CT cerebral angiography.

Predictor	Estimate	Standard error	t- Statistics	p-value
Intercept	27.70	15.036	1.84	0.068
CTDI	1.26	0.512	2.46	0.015
SSDE	-5.67e ^−4^	3.94e ^−4^	-1.44	0.153

## Discussion

This study of 126 participants demonstrated that both patient-related factors and technical parameters significantly influenced contrast enhancement. PET was independently affected by age and gender, while PEA was driven mainly by bolus-tracking thresholds. Arterial enhancement was greater in females and older adults, venous enhancement was strongly linked to PET and blood pressure, and an optimal threshold of 105 ± 5 HU was identified. CTDIvol, but not SSDE, correlated with patient weight, confirming CTDIvol as the more reliable dose metric for cerebral CTA.

These findings highlight that contrast dynamics (PET& PEA) in cerebral CTA are shaped by underlying physiology. The progressive delay in PET with age can be explained by reduced cardiac output, decreased vascular compliance, and slower systemic circulation in older adults. Similar associations between aging and reduced cardiac output have been reported previously. ^16^ In contrast, the shorter PET values in females likely reflect a smaller circulating blood volume and relatively higher cardiac index, leading to faster contrast transit. This is consistent with earlier studies demonstrating that males have 5–10% higher blood volume, which can delay peak enhancement. ^17, 18^ Such physiological differences underscore the need for patient-tailored scan timing rather than a uniform protocol.

These physiological mechanisms, however, are not fully represented by routine bedside measures. In our study, conventional parameters such as heart rate and blood pressure alone were insufficient to account for variability in contrast arrival time, which aligns with prior reports that hemodynamic indices like cardiac output and vascular stiffness are stronger predictors of contrast dynamics. ^19– 21^ Although our sample had more males than females (88 vs. 38), the gender-related differences in PET were consistent and statistically significant, underscoring a genuine physiological basis rather than a sampling artifact. These findings raise the possibility that gender and age should be explicitly considered when designing CTA protocols. ^18^ For example, males and older patients may benefit from slightly delayed scan initiation to ensure optimal arterial opacification, while females and younger patients may not require such adjustments.

Our analysis of arterial enhancement further supports this reasoning. Female gender and older age groups (particularly the two oldest strata) were independently associated with significantly greater arterial opacification. ^20, 22, 23^

PET showed only a borderline trend toward higher enhancement, suggesting that vascular physiology and scan trigger timing interact but that demographic factors exert a more substantial influence on arterial contrast dynamics. Venous enhancement, on the other hand, was strongly linked to PET and blood pressure values, consistent with prior studies demonstrating that delayed arterial arrival or elevated systemic pressures promote earlier venous filling. This overlap between arterial and venous phases narrows the diagnostic window and increases the risk of contamination, which may compromise interpretation, particularly in posterior circulation imaging. ^7, 23^

Identifying the best bolus-tracking threshold of 105 ± 5 HU reinforces the importance of individualized protocol settings. Thresholds that are too high risk delayed triggering and excessive venous overlap, whereas thresholds that are too low may lead to premature acquisition and suboptimal arterial enhancement. Similar to earlier work recommending thresholds in the 100–120 HU range. ^24– 27^ Our findings provide an evidence-based cutoff that balances arterial opacification with minimal venous contamination. This reproducible benchmark is valuable for ensuring consistency across patient populations and reducing the need for repeat scans, thereby improving diagnostic confidence and patient safety.

As an exploratory analysis, we also found that the reliability of HU-based triggering varied across age groups. Thresholds were more predictive in younger patients, whereas performance in older adults was poor. This pattern likely reflects age-related changes in cardiovascular physiology and contrast transit and supports the potential of age-adjusted trigger strategies. Such approaches could further optimize arterial enhancement, minimize venous overlap, and improve the diagnostic accuracy of cerebral CTA, as suggested in recent protocol optimization studies.

Beyond contrast timing, our qualitative analysis of venous enhancement during the angiography phase, assessed using a 3-point Likert scale, ^28^ demonstrated moderate to substantial inter-rater agreement (κ value). This reflects a high level of consistency between the two radiologists and supports the robustness of our qualitative evaluation, ensuring that the imaging results can be interpreted with confidence. Comparable levels of agreement have been reported in previous imaging reproducibility studies, reinforcing the reliability of our findings.

In addition to enhancement dynamics, radiation dose behavior provided further insight into protocol optimization. Understanding radiation dose indices in CTA is essential for balancing diagnostic accuracy with safety, particularly in the context of advanced reconstruction algorithms, where indices may behave differently across anatomical regions. ^29^ We observed a significant positive correlation between CTDIvol and patient weight, whereas SSDE showed no significant correlation. This suggests that phantom-based indices such as CTDIvol remain reliable for head CTA despite increasing emphasis on SSDE in body CT.

The discrepancy with literature favoring SSDE may be explained by anatomical characteristics unique to the head, including relatively uniform skull attenuation and limited variability in patient size. Unlike body CT, where SSDE better reflects tissue attenuation, the rigid cranial structure may buffer size-related differences, ^30, 31^ making CTDIvol a more stable metric for neuroimaging dose assessment. Similar conclusions have been drawn in prior work, and our findings support continued use of CTDIvol in cerebral CTA while encouraging further exploration of patient-specific dose optimization strategies.

Our findings have several important clinical and translational implications. They support the rationale for designing patient-specific CTA protocols that account for age and gender rather than relying solely on fixed timing parameters. Conventional bedside measures such as BP and HR alone proved insufficient to explain contrast dynamics, underscoring the need for more comprehensive cardiovascular assessments in future predictive models. The physiological differences demonstrated here—such as age-related cardiac function decline and blood volume variation between genders provide a plausible framework to interpret enhancement variability and guide protocol optimization.

Clinically, identifying an optimal bolus-tracking threshold of 105 ± 5 HU offers a simple, reproducible adjustment for technologists that minimizes venous contamination and reduces the risk of repeat scans. Recognition of gender and age-related differences in PET highlights the value of individualized scan timing to enhance arterial visualization while avoiding unnecessary contrast or radiation exposure. Finally, our finding that CTDIvol remains a reliable dose metric in head CTA reinforces the practicality of continuing its use for dose monitoring, despite growing advocacy for SSDE in body imaging. Collectively, these insights support the development of patient-tailored CTA protocols that improve diagnostic confidence, enhance safety, and align with broader goals of efficiency and sustainability in neuroimaging practice.

Future research should focus on developing and validating age- and gender specific CTA protocols, especially in relation to contrast injection timing and volume. Comprehensive assessment of cardiac output and vascular compliance may refine predictive models of contrast dynamics. Our findings are based on a fixed-dose, non-weight-adjusted protocol. If lower volumes or weight-adjusted dosing were used, the optimal HU threshold may shift. Future work should test whether the 105 HU cutoff holds under variable injection strategies. Multicenter validation of the 105 HU threshold across different scanners could strengthen generalizability, while exploration of individualized contrast dosing strategies in older adults may enhance safety without reducing diagnostic yield. Finally, larger studies evaluating SSDE in neuroimaging are warranted to clarify its potential role and establish more robust standards for dose optimization.

### Limitations

This study has several limitations. First, the modest sample size and unequal gender distribution (88 males vs. 38 females) may restrict generalizability, although the consistent differences observed across subgroups suggest a genuine physiological basis. Second, the age subgroups were not equally represented, which may reduce the robustness of age-stratified analyses, particularly in the youngest and oldest categories. Third, only basic vital signs were available as cardiovascular surrogates; direct measures such as cardiac output, stroke volume, or vascular compliance were not assessed. Fourth, the findings were derived from a single center using a single scanner platform and a fixed non-weight-adjusted contrast protocol (80 mL at 4 mL/s), which may limit external applicability to other systems or weight-adjusted dosing strategies. The influence of IV access site (arm side, antecubital vs. wrist) was not analyzed, although prior studies suggest this can affect transit dynamics. Fifth, venous enhancement was assessed with a 3-point Likert scale, which, while reproducible, may not capture subtle gradations in venous contamination. Finally, while SSDE was evaluated, its lack of correlation with patient size in head CTA requires confirmation in larger, multicenter cohorts across diverse populations and imaging technologies.

## Conclusion

This prospective study demonstrates that intrinsic patient factors (age and gender) and extrinsic protocol settings (bolus-tracking thresholds) significantly influence arterial opacification and venous contamination in cerebral CTA. Males and older adults exhibited delayed PET, while females and younger patients showed faster contrast transit, underscoring the need for demographic-aware timing strategies. By empirically deriving a bolus-tracking threshold of 105 ± 5 HU that demonstrated only fair discriminatory ability, we propose a pragmatic adjustment that may help balance arterial enhancement with minimal venous overlap and reduce the risk of repeat scans. Contrary to expectations, CTDIvol but not SSDE correlated with patient weight, suggesting that conventional phantom-based indices remain clinically reliable for head CTA dose monitoring. Collectively, these findings support the development of individualized imaging protocols that improve diagnostic consistency, enhance patient safety, and streamline neurovascular imaging workflows.

Beyond clinical relevance, this study contributes to broader global health priorities outlined in the United Nations Sustainable Development Goals (SDGs). By improving diagnostic accuracy while reducing repeat scans, contrast use, and radiation exposure, our findings advance SDG 3 (Good Health and Well-Being) through safer neuroimaging. Identifying the best bolus-tracking threshold and demographic-aware timing strategies reflects SDG 9 (Industry, Innovation, and Infrastructure), while optimizing dose metrics supports SDG 12 (Responsible Consumption and Production). These strategies highlight how protocol optimization in cerebral CTA can improve patient outcomes while fostering more sustainable healthcare practices.

## Ethical considerations

• This prospective observational study was conducted at the Department of Radiodiagnosis and Imaging, Kasturba Hospital, Manipal. With appropriate approvals from the Institutional Ethics Committee (IEC), IEC2: 166/2023. The study was registered with the Clinical Trials Registry of India (CTRI), CTRI/2023/07/054814. Written consent was obtained from all the participants.

## Data Availability

The raw dataset supporting the findings of this study is openly available in Figshare: *Raw dataset for “Optimization of Bolus-Tracking Threshold Levels in Cerebral CT Angiography: Influence of Patient Characteristics on Contrast Enhancement Dynamics and Radiation Dose Metrics.”* Figshare. https://doi.org/10.6084/m9.figshare.30178381 ^32^ The dataset includes:
• Quantitative Hounsfield Unit (HU) measurements obtained from cerebral CT angiography. • Qualitative image quality scores provided independently by two experienced readers. All data have been fully de-identified in accordance with the Safe Harbor method, following HIPAA guidelines ( https://www.hhs.gov/hipaa/for-professionals/privacy/special-topics/de-identification/index.html#standard). The dataset is available under the terms of the Creative Commons Attribution 4.0 International license (CC-BY 4.0), which permits unrestricted use, distribution, and reproduction in any medium, provided the original work is properly cited. This study followed the STROBE (Strengthening the Reporting of Observational Studies in Epidemiology) guidelines for observational studies.
