# Supplementary material for: Optimization of Bolus-Tracking Thresholds levels in Cerebral CT Angiography: Influence of Patient Characteristics on contrast enhancement dynamics and radiation dose metrics
Source: F1000Res. 2025 Nov 29;14:1219. Originally published 2025 Nov 6. [Version 2] doi: 10.12688/f1000research.171062.2 (PMC12891954; doi:10.12688/f1000research.171062.2)
Supplement: Peer review history

# Reviewer report: Reviewer response for version 2

**DOI:** 10.5256/f1000research.192337.r447835

**Published:** 2026-02-10

**Authors:** Dolly Sharma

**Affiliations:** 1 Radio imaging Technology, Shree Guru Gobind Singh Tricentenary University (Ringgold ID: 389839), Gurugram, Haryana, India; 2 Charotar University of Science and Technology, Gujarat, India

This study presents a well-designed and clinically relevant evaluation of bolus-tracking optimization in cerebral CT angiography. The authors effectively address a common practical challenge—venous contamination—by integrating patient-related physiological factors with enhancement dynamics and radiation dose metrics. The prospective methodology, adequate sample size, and use of blinded radiologist assessment strengthen the validity of the findings. The analysis clearly demonstrates how intrinsic factors such as age and gender significantly influence peak enhancement time and arterial attenuation, underscoring the need for individualised CTA protocols. The identification of an optimal bolus-tracking threshold of 105 ± 5 HU, supported by ROC analysis, provides a practical and implementable recommendation that can improve arterial visualisation while minimising venous overlap, particularly in younger patients. Additionally, the clarification of CTDIvol as a more reliable dose index than SSDE in head CTA adds value for protocol standardisation. Overall, the study offers meaningful insights that enhance image quality, promote dose awareness, and align with sustainable and patient-centred imaging practices.

Is the work clearly and accurately presented and does it cite the current literature?

Yes

If applicable, is the statistical analysis and its interpretation appropriate?

Yes

Are all the source data underlying the results available to ensure full reproducibility?

Yes

Is the study design appropriate and is the work technically sound?

Yes

Are the conclusions drawn adequately supported by the results?

Yes

Are sufficient details of methods and analysis provided to allow replication by others?

Yes

Reviewer Expertise:

Medical Imaging Technology including Magnetic Resonance Imaging, Computed tomography, Ultrasound, radiography and X-ray studies.

I confirm that I have read this submission and believe that I have an appropriate level of expertise to confirm that it is of an acceptable scientific standard.

# Reviewer report: Reviewer response for version 1

**DOI:** 10.5256/f1000research.188606.r431358

**Published:** 2025-11-25

**Authors:** Manna Debnath

**Affiliations:** 1 Pramukhswami Medical College and Shree Krishna Hospital, Bhaikaka University, Anand, Gujarat, India

Reviewer comments:

1. **The introduction is well written.**
2. **In Table 1, please include the mAs parameter under the scan protocol section.**
3. **In Table 2, the height is reported as 164 ± 9.75, while in the paragraph above, it appears as 164 ± 9.8. Although the values are nearly the same, please ensure the exact number is used consistently in both places to avoid confusion for readers.**
4. **The mean equals the median only for a symmetrical distribution. In the Results section (first paragraph), I suggest replacing the term “median” with “mean.”**
5. **The discussion and conclusion are well written.**

Is the work clearly and accurately presented and does it cite the current literature?

Yes

If applicable, is the statistical analysis and its interpretation appropriate?

Yes

Are all the source data underlying the results available to ensure full reproducibility?

Yes

Is the study design appropriate and is the work technically sound?

Yes

Are the conclusions drawn adequately supported by the results?

Yes

Are sufficient details of methods and analysis provided to allow replication by others?

Yes

Reviewer Expertise:

Medical Imaging Technology

I confirm that I have read this submission and believe that I have an appropriate level of expertise to confirm that it is of an acceptable scientific standard.

# Response

**Published:** 2025-11-26

**Authors:** Abhimanyu Pradhan

**Affiliations:** Medical Imaging Technology, Manipal College of Health Professions, Manipal, Karnataka, India

The current version of this article incorporates several revisions made in response to reviewer feedback and internal review.

 First, the scan protocol section has been updated to include the mAs parameter, providing a more detailed and transparent outline of the imaging methodology.

 Second, minor inconsistencies in the reporting of participant characteristics have been corrected; specifically, the height value has been standardized to 164 ± 9.75 across both the text and Table 2 to ensure accuracy and uniformity.

 In addition, we clarified the statistical reporting in the Results section by explicitly stating that non-normally distributed variables are presented as median (IQR), thereby aligning the text with the appropriate statistical conventions.
